# Training a Fit-For-Purpose Rural Health Workforce for Low- and Middle-Income Countries (LMICs): How Do Drivers and Enablers of Rural Practice Intention Differ Between Learners From LMICs and High Income Countries?

**DOI:** 10.3389/fpubh.2020.582464

**Published:** 2020-10-19

**Authors:** Karen Johnston, Monsie Guingona, Salwa Elsanousi, Jabu Mbokazi, Charlie Labarda, Fortunato L. Cristobal, Shambhu Upadhyay, Abu-Bakr Othman, Torres Woolley, Balkrishna Acharya, John C. Hogenbirk, Sarangan Ketheesan, Jonathan C. Craig, Andre-Jacques Neusy, Sarah Larkins

**Affiliations:** ^1^Anton Breinl Research Centre of Health Systems Strengthening, College of Medicine and Dentistry, James Cook University, Douglas, QLD, Australia; ^2^Ateneo de Zamboanga University School of Medicine, Zamboanga City, Philippines; ^3^University of Gezira Faculty of Medicine, Gezira, Sudan; ^4^School of Medicine, Walter Sisulu University, Mthatha, South Africa; ^5^School of Health Sciences, University of the Philippines, Manila, Philippines; ^6^Patan Academy of Health Sciences, Patan, Nepal; ^7^Centre for Rural and Northern Health Research, Laurentian University, Sudbury, ON, Canada; ^8^College of Medicine and Public Health, Flinders University, Adelaide, SA, Australia; ^9^Training for Health Equity Network, New York, NY, United States

**Keywords:** rural practice intention, rural medical practice, barriers and enablers, rural practice, human resources for health (HRH), LMIC = low- and middle-income countries, practice intentions, social accountability

## Abstract

Equity in health outcomes for rural and remote populations in low- and middle-income countries (LMICs) is limited by a range of socio-economic, cultural and environmental determinants of health. Health professional education that is sensitive to local population needs and that attends to all elements of the rural pathway is vital to increase the proportion of the health workforce that practices in underserved rural and remote areas. The Training for Health Equity Network (THEnet) is a community-of-practice of 13 health professional education institutions with a focus on delivering socially accountable education to produce a fit-for-purpose health workforce. The THEnet Graduate Outcome Study is an international prospective cohort study with more than 6,000 learners from nine health professional schools in seven countries (including four LMICs; the Philippines, Sudan, South Africa and Nepal). Surveys of learners are administered at entry to and exit from medical school, and at years 1, 4, 7, and 10 thereafter. The association of learners' intention to practice in rural and other underserved areas, and a range of individual and institutional level variables at two time points—entry to and exit from the medical program, are examined and compared between country income settings. These findings are then triangulated with a sociocultural exploration of the structural relationships between educational and health service delivery ministries in each setting, status of postgraduate training for primary care, and current policy settings. This analysis confirmed the association of rural background with intention to practice in rural areas at both entry and exit. Intention to work abroad was greater for learners at entry, with a significant shift to an intention to work in-country for learners with entry and exit data. Learners at exit were more likely to intend a career in generalist disciplines than those at entry however lack of health policy and unclear career pathways limits the effectiveness of educational strategies in LMICs. This multi-national study of learners from medical schools with a social accountability mandate confirms that it is possible to produce a health workforce with a strong intent to practice in rural areas through attention to all aspects of the rural pathway.

## Introduction

Equity in health outcomes for rural and remote populations in low- and middle-income countries (LMICs) is limited by a range of socio-economic, cultural and environmental determinants of health. Access to comprehensive primary health care services provided by a well-trained and fit-for-purpose health workforce in rural and remote areas is one important strategy to address health disparities (1, 2). LMICs are grappling with overall shortages of human resources for health (HRH), with the World Health Organization calculating a global shortage of 17.4 million in 2013, projected to decrease to 14.5 million by 2030 [based on an indicative aggregate density threshold of doctors, nurses and midwives of 4.45 per 1,000 population; (3)]. These shortages are in turn inequitably distributed, with the most pronounced shortages in countries with the least resources. In many LMICs there is a long-term, embedded underinvestment in education and training of the health workforce, despite evidence of the economic benefits of this investment (4). This issue is exacerbated by limited communication between the education and training sector and the health sector, in terms of ensuring that the competencies of graduating health workers are appropriate to meet the needs of the population they serve (3).

In addition to these absolute shortages in HRH, in almost all parts of the world, the health workforce is geographically and vocationally mal-distributed, with a relative over-supply in the urban centers, and health workforce shortages in rural and remote areas, especially in areas that rate lower on livability scales (3). There are many reasons for this mal-distribution. For example, rural and primary care practice are often perceived as “second class” options—a misperception that is inadvertently and perhaps deliberately reinforced because, particularly in medicine, most health professional students are the children of the urban elite, most training takes place in large urban institutions, and the most visible role models during this training are city-based specialists (5). In addition, especially in medicine, the “hidden curriculum” and remuneration structures within health care reward sub-specialization and procedural work over generalism. Together with often under-developed and under-resourced postgraduate training programs for primary care, this may make primary care or general practice unnecessarily challenging and unjustly unpopular as a career option for medical graduates (2).

Health professional education that is sensitive to local population needs and that attends to all elements of the rural pathway is vital to increase the proportion of the health workforce that practices in underserved rural and remote areas (6). In practice, this means paying close attention to: (i) rurally-oriented selection processes; (ii) rural and primary health care-oriented curriculum delivered largely in rural and remote locations; (iii) rural and regional postgraduate training pathways and support; and (iv) ensuring exposure to a wide range of appropriate rural and remote mentors and teachers (6–8).

The Training for Health Equity Network (THEnet; www.thenetcommunity.org), is a community-of-practice of 13 health professional education institutions from 10 countries in high, middle and low income settings, with a clear, self-identified mandate to deliver socially accountable education to produce a fit-for-purpose health workforce and contribute to Universal Health Care (UHC). One of the earliest tasks of the THEnet Evidence Group was to collaboratively develop and test a common Evaluation Framework to be used by health professional schools to critically self-evaluate the degree to which they were achieving their own social accountability goals (9). Part of this work is holding ourselves, as educators, responsible for where our graduates go, what they do, and the degree to which practice location and skill set match need. Since 2010, THEnet has been coordinating an international graduate outcome study, to look at the associations between learner characteristics, intentions to practice and actual practice location and discipline in these differing contexts and now has data on more than 6,000 learners from both LMICs and high income countries (HICs).

This study is part of a series of multi-institutional collaborative research supported by THEnet and its institutional partners to gather evidence on the outcomes and impact of socially accountable health professional education(SAHPE), using the THEnet's Framework for Socially Accountable Health Workforce Education as a logicmodel [https://thenetcommunity.org/the-framework/; (9–11)]. Collaborative research between THEnet partner schools is helping to demonstrate the success of a socially accountable approach (7, 12–14). This manuscript considers learner characteristics, considers learner characteristics and country contextual factors associated with: (i) intention to practice in rural and underserved areas; (ii) intended discipline of practice; and (iii) intention to emigrate at graduation, and analyses how these associations vary between LMICs and HICs.

## Methods

The THEnet Graduate Outcome Study (GOS) is an international prospective cohort study, now with more than 6,000 learners from nine health professional schools in seven countries [included in these analyses four LMICs; the Philippines (two schools), Sudan, South Africa and Nepal, and two HIC; Australia and Canada]. Surveys of learners are administered at entry to medical school, at exit from medical school and at years 1, 4, 7, and 10 thereafter. Underserved populations are defined in terms of three dimensions: (i) geographic factors; (ii) socioeconomic factors; and (iii) socio-cultural disadvantage (due to religion, caste, minority ethnicity or status as a refugee or recent immigrant). Rurality is defined contextually in terms of quintiles for each country that mirror population quintiles as closely as possible. Further details of the methodology are available elsewhere (12, 13). Longitudinal matching of entry and exit surveys is now possible for increasing numbers of learners and we are building up the longitudinal cohort into the postgraduate years which allows correlation with actual practice in time, and the inclusion of data about clinical placements. Different schools have commenced data collection for the GOS in different calendar years according to when ethical approval was granted.

Building on earlier work, in this analysis we consider the association of intention to practice in rural and other underserved areas and a range of individual and institutional level variables at two time points—entry to and exit from the medical program; that is cross-sectional data at two time-points. At each point, Pearson χ^2^ tests and binary adjusted logistic regression are used to examine the individual and institutional factors associated with: (i) intention to practice in rural areas; (ii) intention to practice in generalist disciplines; and (iii) intention to emigrate. Longitudinal paired data analysis used McNemar's test. These findings are then triangulated with a sociocultural exploration of the structural relationships between educational and health service delivery ministries in each setting, status of postgraduate training for primary care, current policy settings with regard to HRH planning and support, and other relevant factors that may influence support. This concurrent, mixed methods design (15) was constructed to optimize our understanding of the factors that influence HRH outcomes in medical school settings. Data to inform this analysis come from document review of publicly available sources and websites, supplemented by direct communication between authors; all embedded experts in health professional education in their own contexts. To further the trustworthiness of our results we also triangulated findings with those from complementary research from within the THEnet community, to understand the factors contributing to these strong drivers (14, 16).

Ethics approval was received from the ethics review committees of each participating school. Individual informed written consent was obtained from all participants.

## Results

Of the 6,000 learners enrolled in the study, findings presented here include data from 3,849 learners at entry and 1,229 learners at exit. Of these learners, 149 provided data at entry and exit with 45% of learners with such matching data having attended school in a LMIC. The analyses involve survey data from medical schools in LMICs, including 864 learners from Gezira University in Sudan, and 665 learners from Walter Sisulu University in South Africa, both specifically established to meet the needs of the rural and impoverished regions in their respective countries. We also have data from 382 learners across two medical schools in the Philippines. Ateneo de Zamboanga University in Mindanao, which is founded on a strong social mission to meet the needs of rural and underserved populations across Mindanao in Southern Philippines; and the School of Health Sciences, University of the Philippines, Leyte, which provides a stepladder curriculum to meet the needs of populations in the Philippines archipelago. Entry level data from two cohorts of students from Patan Academy of Health Sciences in Nepal, established with a specific mission to meet the health needs of the dispersed rural populations of Nepal are also available. Comparison data for rural practice intention are available for HIC schools in Australia (James Cook University and Flinders University; both with a social mandate to meet the needs of rural and remote populations, and Australian Aboriginal and Torres Strait Islander populations) and Canada (Northern Ontario School of Medicine; established to meet the health needs of rural, Indigenous, Francophone and the general population of Northern Ontario).

The demographics of learners at these schools differ from those of most medical schools, with higher proportions of students from rural and remote backgrounds (40.4%), from low socioeconomic and educational backgrounds (28.9% from low income background, 34.4% neither parent completed university, 29.4% spent more than 4 years in public schooling), and from underserved population groups (23.9%; Table 1).

**Table 1:** Demographic profile and background characteristics for participating THEnet schools.

**Mean age (SD)**		**Female *n*/*N* (%)**	**Lowest two quintiles of income (background) *n*/*N* (%)**	**Identify as underserved population *n*/*N* (%)**	**Neither parent attended university *n*/*N* (%)**	**Years of public schooling (>4 years) *n*/*N* (%)**	**Rural background 1-3^a^*n*/*N* (%)**
Entry *n* = 3,851 21.14 (4.34)	Exit *n* = 1,187 26.33 (4.12)	2,917/4,915 (59.3)	863/2,987 (28.9)	984/4,121 (23.9)	1,555/4521 (34.4)	1,336/4,538 (29.4)	1,613/3,989 (40.4)

a *Rural quintiles (1, remote village; 2, small rural town; 3, large rural town) vs. Urban quintiles (4, major regional center and 5, major city or capital city). Learners with primary school background in a country other than the country where they attended medical school were excluded from this variable. Most schools used population size to define quintiles; NOSM and UPSHS based quintiles on government socioeconomic classifications*.

Contextual information about these health professional courses, their structure and setting and their relationships with local health services and training programs in the region are summarized in Table 2. The schools have all clearly evolved to serve underserved populations in their regions. Notable contextual features include the differences between LMICs and HICs in terms of recognition and strength of training programs for family medicine/general practice as a specialty. Australia and Canada both have strong, certified training programs for general practice/family medicine, with recognition of the important role played by these practitioners in providing first contact, comprehensive continuing care and acting as a gatekeeper for access to the specialist system. Despite the efforts of THEnet partners and a family practice program delivered by PAHS, both Nepal and the Philippines have comparatively limited recognition, training or support for primary care and patients are able to self-refer for specialist care (Table 2). To some extent, this is still the case in Sudan, though progress in training for primary care has been made through establishment of a community-oriented postgraduate training program in family medicine in 2010. South Africa has a strong training program for family medicine, but lack of clarity in policy about the role of primary care providers within the health system has limited their effectiveness in working as part of a team in the community to deliver primary health care services.

**Table 2:** Contextual information about these health professional courses, their structure and setting and their relationships with local health services.

**Country**	Medical education context^*^	**Relationship between health system and educational planning**	**Health professional school, foundation year, (year of joining graduate outcome study)**	**Training structure (size of entry cohort of medical students in 2013)**	**Priority population**	**Participants *n* (response rate, %)**
The Philippines	Population density 358 people/km^2^ Gross national income per capita $7290 (2012) 40 medical schools Physician density 1.3/1,000 (2010) Poor recognition of general practice/primary care as a specialty, but many graduate with public health qualifications. Weak US-style family medicine training and certification	Historically poor coordination between health professional education and health systems Patients can self-refer to specialists, bypassing primary care. Strong and largely unregulated influence of pharmaceutical sector Largely privatized higher education system, and large wage disparities between public and private systems for healthcare workers Maldistribution - <10% of graduates serve rural areas Health training as an export industry - high rates of medical and nursing emigration	Ateneo de Zamboanga University School of Medicine, (AdZU) Zamboanga City, Mindanao. 1993 (2013) School of Health Sciences, University of the Philippines, (SHS) Palo, Leyte. 1976 (2013)	Four-year graduate MD training, about 50% community based. One year internship, 50% in rural health units, emergency and district hospitals (48 students) Five-year graduate MD program. Multilevel entry stepladder curriculum. Six months in Year 2 and all of Year 5 in rural community practice setting Also trains community workers/midwives and nurses)(15 students)	Rural underserved areas of Mindanao, especially Zamboanga peninsular and outlying islands Rural underserved populations in the central Philippines Indigenous peoples	Entry 216 (87.4) Exit 150 (84.7) Entry 33 (89.2) Exit 50 (72.5)
Sudan	Population density 25 people/km^2^ Gross national income per capita $3220 (2012) 29 medical schools (8 private) Physician density 0.3/1000 (2017) Role of primary care in health system underdeveloped and undervalued in health system Two year community-oriented postgraduate training in family medicine developed in partnership with Gezira Ministry of Health	Four older medical schools, then rapid proliferation of new schools mostly in Khartoum. Perceived decline in training standards Widespread emigration of health professionals for social and economic reasons In last decade partnerships between education institutions, Ministry of Health and Education to progress training for primary health care, including an initiative through U Gezira (17) Feminisation of medical workforce caused issues in rural coverage and workforce (18)	University of Gezira Faculty of Medicine, Gezira State. 1975 (2013)	Five-year undergraduate training program Twenty percent of time allocated for community-based education (270 students)	Rural underserved areas in Gezira region	Entry 805 (66.6) Exit 59 (29.6%)
South Africa	Population density 48 people/km^2^ Gross national income per capita $11,970 (2012) Nine medical schools Physician density 0.9/1,000 (2017) Four year postgraduate community-based training program (UK/Aust style) for family medicine—specialist recognition	Previously limited coordination between HRH training and deployment with no integrated data source for HRH planning, despite HRH making up almost 2/3 of public health expenditure. Previous planning efforts not implemented Absolute shortages in HRH, especially beyond urban centers, and in public sector, with high professional emigration	Walter Sisulu University Faculty of Health Sciences (WSU) Mthatha, South Africa. 1985 (2013)	Six year undergraduate program, rural experiences in Years 1–3 and 6 months in Year 5 Also trains Clinical Associates (PAs) (120 students)	Rural underserved areas of Eastern Cape and KwaZulu Natal provinces	Entry 563 (91.4%) Exit 102 (58%)
	Tension between health policy focused on public PHC (without a clear role for family physicians) and health system with strong specialist and hospitalist focus	Introduction of National Health Insurance has spurred more coordinated efforts and integrated planning through the NHI Fund, although still in its infancy (19, 20)				
Nepal	Population density 196 people/km^2^ Gross national income per capita $2,170 (2012) 19 medical schools (15 private) Physician density 0.75/1,000 (2018) Three year postgraduate medical training program in general practice to address rural doctor shortage Lack of well-defined career pathway for general practice with limited ability to serve the rural population or strengthen PHC approach due to health system factors that favor speciality practice (21)	Poor staff performance in terms of productivity, quality, availability, and competency Fragmented approach to HRH planning, management, and development Imbalance between supply and demand, and narrow skill mix Limited HRH financing Low attraction/retention in public service, and “brain drain” largely due to the migration of health workers (22)	Patan Academy of Health Sciences (PAHS) Patan, Nepal 2008 (2019)	Five year undergraduate problem-based learning curriculum. Not-for-profit institution. Adapted for local priority issues and priorities. Selective recruitment prioritizing rural students and extensive rural community placements (65 students; 2019)	Rural underserved areas, the poor and diverse ethnic groups, particularly those in northern and Western Nepal	Entry 130 (100%)
Australia	Population density 3 people/km^2^ Gross national income per capita $41,590 22 medical schools Physician density 3.7/1,000 (2017) Strong postgraduate training program (3–4 years) for general practice with independent certification exams General practitioners and “rural generalists” have well-recognized role as gatekeepers and work in private practices, community health centers, rural hospitals and community-controlled health services	Well supplied in terms of numbers of doctors and nurses but ongoing problems with vocational (insufficient generalist) and geographical maldistribution Various incentives and policies introduced to address these with variable success Separate Ministry for Health and Education, but relatively cohesive and functional mechanisms to create joint planning—e.g., Medical Training Review Panel (23) Reducing earlier reliance on international medical graduates Attention to entire rural pathway demonstrated to produce successful outcomes	James Cook University College of Medicine and Dentistry (JCU) Townsville, Queensland 2000 (2013) College of Medicine and Public Health (FU) Adelaide, South Australia. 1995 (2013)	Six year undergraduate MBBS program, entirely regional, including 20 weeks in small rural and remote settings Also trains dentists and Physician Assistants (238 students) Four year graduate program based in Adelaide or in Darwin. Option for 1 year Parallel Rural Curriculum (30 students) (160 students)	Rural, remote, Aboriginal and Torres Strait Islander populations, and others in tropical Australia Rural, remote and Aboriginal and Torres Strait Islander populations	Entry 1,367 (83.1%) Exit 509 (42.0%) Entry 480 (74.2) Exit 167 (57.5)
Canada	Population density 4 people/km^2^ Gross national income per capita $41,170 (2012) 17 medical schools Physician density 2.3/1,000 (2016) Family medicine is a strong, recognized specialty and gatekeeper to specialist care School based family medicine programs with defined curriculum and an end-point examination	HRH comprise a large part of health expenditure Strong system of universal access and coverage through family practice, rural hospitals and regional/urban hospitals Parallel private health system Limited processes to track predicted actual and predicted health workforce over time at national level (although some local initiatives) and some national descriptive data.	Northern Ontario School of Medicine (NOSM) Thunder Bay and Sudbury, Canada. 2005 (2016)	Four year graduate program. Entirely regional. Twelve weeks Indigenous and rural community placements plus 8 month community longitudinal integrated clerkship (64 students)	Rural, Indigenous, Francophone and general population of Northern Ontario	Entry 255 (99.2%) Exit 192 (98.5)

* *From World Health Organization Global Health Observatory (http://apps.who.int/gho/data/node.country) and World Bank (https://data.worldbank.org/indicator/). Most recent available data point used*.

### Intention to Practice in Rural Areas

At entry to medical school, a high proportion of learners intended to practice in rural areas (defined as a remote, small rural or large rural town; quintiles 1–3) after completing postgraduate training (1,864/3,598; 51.8%). In these cross-sectional samples across all these schools, a significantly smaller proportion of learners intended to practice in rural areas at exit than at entry (502/1,135, 44.2%; OR 0.74, 95% CI 0.65–0.84, *p* < 0.001). Importantly, there was no significant change in the proportion of learners intending to practice in rural areas between entry and exit for the 144 learners with matching data (*p* = 0.644).

Binary logistic regression analyses adjusting for confounders showed that learner characteristics and school being located in a LMIC were significantly associated with intention to practice in a rural area, and these differed at entry and exit (Tables 3, 4). Learners with a rural background (where the majority of primary schooling was completed in a remote, small rural or large rural town; quintiles 1–3) were 3.3 times more likely to intend to practice in rural areas at entry (*p* < 0.001; Table 3). Similarly, learners with a lower income background (defined as parental income in the bottom two quintiles) and those who identified as a sociocultural underserved group were 1.7 times (*p* < 0.001) and 1.3 times (*p* = 0.04) more likely to intend to practice in rural areas at entry, respectively (Table 3). In comparison, at exit, a higher likelihood of intention to practice in a rural area was associated with attending medical school in a LMIC (AOR 2.01, *p* < 0.001), being female (AOR 1.80, *p* = 0.001) and having a rural background (AOR 1.89, *p* < 0.001), with a weak association with age (Table 4).

**Table 3:** Predictors of intention to work in a rural location where binary variable is rural vs. urban location at entry^a^.

	**Number in unadjusted analysis**	**Unadjusted odds ratios (95% CI; *p*-value)**	**Adjusted odds ratios (95% CI; *p*-value) *N* = 1,574**
Increasing age	3,573	1.03 (1.01–1.04; 0.001)	1.01 (0.98–1.04; 0.48)
LMIC school	3,598	0.87 (0.76–0.99; 0.033)	0.80 (0.63–1.00; 0.051)
Female	3,592	1.26 (1.10–1.44; 0.001)	1.22 (0.82–1.51; 0.073)
Income bottom two deciles	2,169	1.86 (1.54–2.26; <0.001)	1.66 (1.29–2.13; <0.001)
Identify as underserved group	3,063	1.90 (1.61–2.25; <0.001)	1.32 (1.02–1.72; 0.04)
Rural background (Quintiles 1, 2 and 3)	2,895	3.45 (2.94–4.04; <0.001)	3.29 (2.63–4.11; <0.001)

a *Rural quintiles (1, remote village; 2, small rural town; 3, large rural town) vs. Urban quintiles (4, major regional center and 5, major city or capital city). Excludes learners with an international background. CI, confidence interval*.

**Table 4:** Predictors of intention to work in a rural location where binary variable is rural vs. urban location at exit^a^.

	**Number in unadjusted analysis**	**Unadjusted odds ratios (95% CI; *p*-value)**	**Adjusted odds ratios (95% CI; *p*-value) *N* = 597**
Increasing age	1,102	1.07 (1.04–1.10; <0.001)	1.09 (1.04–1.14; 0.001)
LMIC school	1,135	1.50 (1.16–1.93; 0.002)	2.01 (1.47–3.00; <0.001)
Female	1,132	1.51 (1.18–1.93; 0.001)	1.80 (1.26–2.55; 0.001)
Income bottom two deciles	790	1.09 (0.80–1.50; 0.576)	0.85 (0.57–1.25; 0.407)
Identify as underserved group	974	1.15 (0.83–1.58; 0.407)	0.80 (0.51–1.25; 0.335)
Rural background (Quintiles 1, 2 and 3)	958	1.76 (1.36–2.29; <0.001)	1.89 (1.33–2.68; <0.001)

a *Rural quintiles (1, remote village; 2, small rural town; 3, large rural town) vs. Urban quintiles (4, major regional center and 5, major city or capital city). Excludes learners with an international background. CI, confidence interval*.

The rural quintile of learners' background was strongly associated with intent to practice in rural areas, at entry and exit (Pearson's χ^2^ for trend; 281.920, df = 4, *p* < 0.001; 43.035, df = 4, *p* < 0.001, respectively). This trend was observed even after adjusting for age, school's country income setting, gender, family income and sociocultural underserved group. Compared with learners with a metropolitan background, learners from small rural towns had the highest likelihood of intention to practice in rural areas at entry (AOR 6.32, *p* < 0.001) and exit (AOR 3.82, *p* < 0.001), followed by learners from remote towns, then regional centers, then major urban centers (Supplementary Materials 1, 2).

Learners with an international background (who completed the majority of primary schooling in a different country to their medical school) were less likely than learners with a domestic background to have rural practice intentions at entry (*N* = 3,500; Pearson χ^2^ 60.217, df = 1, *p* < 0.001) and exit (*N* = 516; Pearson χ^2^ 10.849, df = 1, *p* = 0.001).

### Intended Discipline of Practice

**Figure 1:**
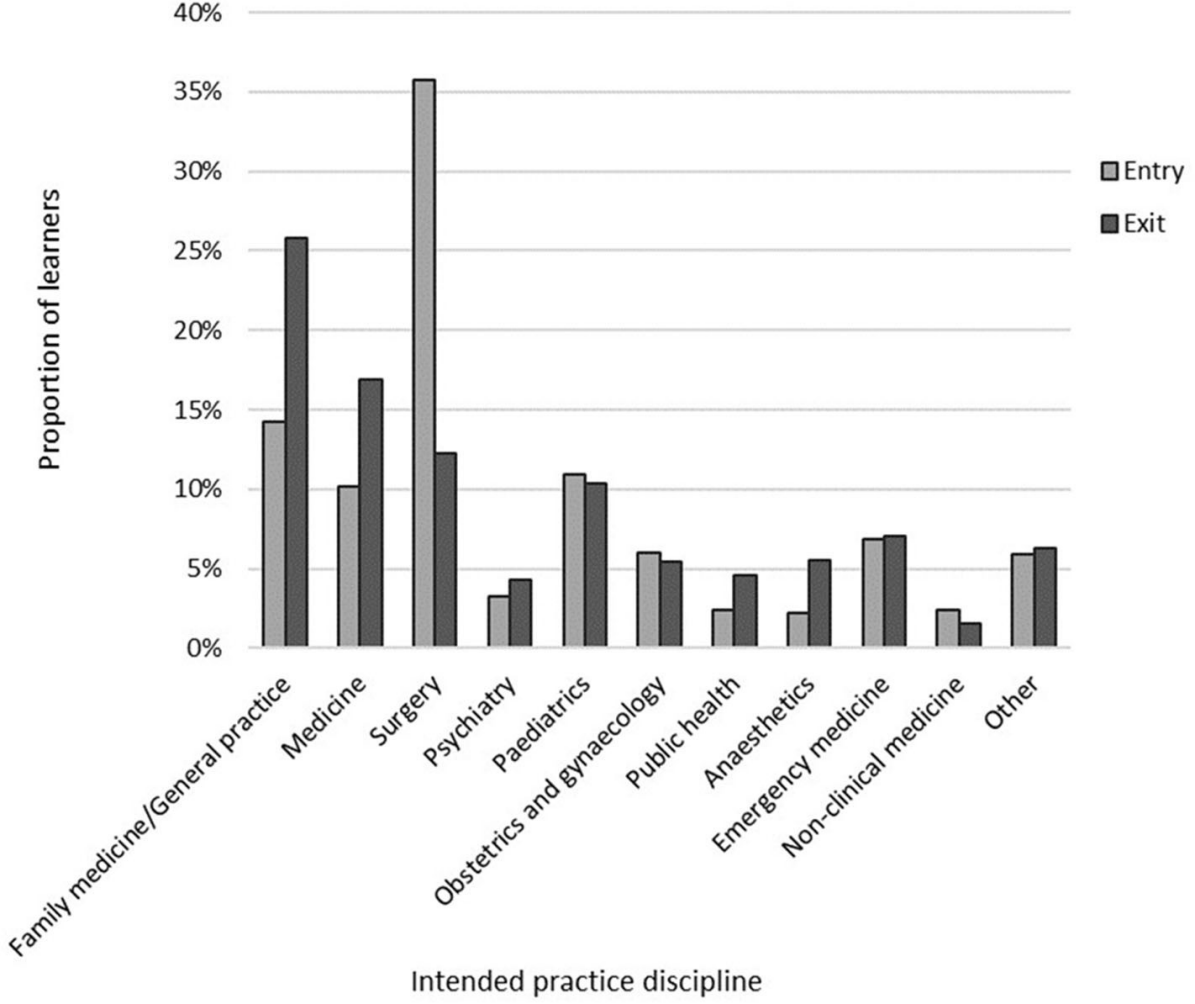
Practice discipline intentions at entry to and exit from medical school.

For the cross-sectional data, learners were twice as likely to intend to practice in family medicine/general practice at exit (282/1,093, 25.8%) compared with entry (412/2,892, 14.2%; OR 2.09, 95% CI 1.76–2.48, *p* < 0.001; Figure 1). Learners were also significantly less likely to have practice intentions in the discipline of surgery at exit (134/1,093, 12.3%) than at entry (1,033/2,892, 35.7%; OR 0.25, 95% CI 0.21–0.31, *p* < 0.001). For the learners with paired data, there was no significant change in the proportion of learners who intended to work in family medicine/general practice between entry and exit (*p* = 0.664). There was, however, a significant change in the proportion intending to practice in surgery with 23/31 (74.2%) changing away from surgical intent between entry and exit (*p* < 0.001).

Binary logistic regression analyses showed that age, gender, high income setting and rural background were associated with learners' intention to practice in family medicine/general practice (Supplementary Material 3). Females were twice as likely as males to have practice intentions in generalist disciplines at exit, but not at entry (exit AOR 2.10, *p* = 0.002; entry AOR 0.93, *p* = 0.667). Learners with a rural background were twice as likely to intend generalist practice as learners with an urban background, at entry (AOR 2.05. *p* < 0.001), but not at exit (AOR 1.51, *p* = 0.063). At entry and exit, learners from schools in LMIC were much less likely to intend to practice in generalist disciplines than learners in HIC (entry AOR 0.19, *p* < 0.001; exit AOR 0.15, *p* < 0.001).

### Intention to Work Abroad

A significantly smaller proportion of learners at exit reported an intention to practice abroad than at entry (exit 376/778, 48.3%; entry 1,591/2,459, 64.7%; OR 0.51, 95% CI 0.43–0.60, *p* < 0.001). Interestingly, the proportion of learners with matching entry and exit data who changed their intention from working abroad to an intention to stay in their country (18/31, 58.0%) was significantly greater than the proportion of learners who changed their intention to instead work abroad (7/47, 14.9%; *p* = 0.043).

Learners with an international background were significantly more likely than learners with a domestic background to have intentions of working abroad at entry (both *p* < 0.001). After adjusting for age, school's country income setting, gender, family income background and sociocultural underserved group, learners with an international background were 4.4 times more likely to intend to work abroad at exit, than learners with a domestic background (95%CI 2.01–9.55, *p* < 0.001). However, reasons for emigration vary; of the 76 learners at exit with an international background, 20 intended to work abroad because their country needs doctors (*n* = 6) or to stay close to home (*n* = 14).

Binary logistic regression analyses of learners with a domestic background showed that decreasing age, not identifying as an underserved group and having an urban background were associated with an intent to practice abroad at entry (AOR 0.90, *p* < 0.001; AOR 2.31, *p* < 0.001; AOR 1.44, *p* = 0.007; respectively; Supplementary Material 4). At exit, decreasing age was associated with higher intent to work abroad (AOR 0.85, <0.001; Supplementary Material 5).

When restricting the analysis to learners from LMIC, where emigration of trained HRH is a major concern, the proportion of learners with an intention to practice abroad was significantly smaller at exit (75/256, 29.3%), than at entry (745/1,203, 61.9%; OR 0.25, 95% CI 0.19–0.34, *p* < 0.001). The proportion of learners who intended to work abroad for more than 10 years was also significantly smaller at exit, than at entry (exit 4/50, 8.0%; entry 109/413, 26.4%; OR 0.24, 95% CI 0.09–0.69, *p* = 0.005^FET^). After adjusting for confounding variables, decreasing age was the only learner characteristic that was significantly associated with intention to practice abroad at entry and exit (entry AOR 0.81, *p* < 0.001; exit AOR 0.53, *p* < 0.001; Table 5).

**Table 5:** Predictors of intention to work abroad where binary variable is “yes—intend to work abroad” and “No—don't intend to work abroad” at entry and exit for schools in LMIC.

	**Entry**	**Exit**
	**Adjusted odds ratios (95% CI;** ***p*****-value) (*****N*** **=** **560)**	**Adjusted odds ratios (95% CI;** ***p*****-value) (*****N*** **=** **176)**
Increasing age	0.81 (0.77–0.86; <0.001)	0.53 (0.41–0.70; <0.001)
Female	0.69 (0.48–1.01; 0.055)	1.45 (0.60–3.54; 0.413)
Income top two deciles	2.31 (1.36–3.93; 0.002)	2.74 (0.95–7.90; 0.063)
Does not identify as underserved group	1.82 (1.23–2.69; 0.003)	1.37 (0.49–3.84; 0.547)
Urban background (Quintiles 4 and 5)	1.85 (1.26–2.73; 0.002)	1.78 (0.68–4.64; 0.240)

*Excludes learners with an international background. CI, confidence interval*.

*Unsure option removed from analysis*.

For all learners combined, the main motivation to work abroad, at entry and exit, was to gain experience (entry 783/1,513, 51.8%; exit 174/360 48.3%). A much higher proportion of learners in LMIC were motivated to work abroad for this reason at exit, compared with learners in HIC (LMIC 50/72, 69.4%; HIC 124/288, 43.1%). In LMIC, an intention to stay at home rather than work abroad at exit was motivated by a desire to respond to the need for doctors in their home country (91/165, 55.2%) or a preference to stay close to home or family (66/165, 40.0%).

## Discussion

This multi-national study of learners from medical schools with a social accountability mandate confirms that it is possible to produce a health workforce with a strong intent to practice in rural areas, in both LMIC and HIC settings through attention to all aspects of the rural pathway. These same learners express a strong (and increasing) intention to practice in generalist disciplines or primary care, and a decreasing intention to work abroad following graduation. These trends are particularly strong for learners from rural and low socio-economic backgrounds, and in contexts where there are clear roles for primary care providers in the rural health system, with a strong training program and postgraduate support. It follows, that to strengthen the rural health workforce in LMICs, it is important to increase the number of health professional education institutions with a social mission that are located in regional and rural areas, that recruit learners from rural backgrounds, and deliver primary health care focused curricula largely in regional, rural and community settings. As Strasser and Neusy (6) point out, training future health professionals in the contexts in which they will later be needed to serve is essential. In addition, it is vital to use policy levers to clarify the role of primary care providers/family physicians in the health system, and work to increase the prestige and recognition of these roles within the health system (24). Building postgraduate training programs with certification exams in family medicine/general practice, so that it can be recognized as a specialty in its own right, is an important step along that journey (17, 25).

Previous published analyses (based on smaller numbers of learners) demonstrated an association between students' intent to practice in a rural location after completing postgraduate training, and the drivers of: (i) coming from a rural and/or low income background; and (ii) their medical school being located in a regional area (13). This analysis confirmed the association of rural background with an intention to practice in rural areas at both entry and exit, and furthermore, found a consistent trend of higher odds of rural practice intention associated with increasing remoteness of learners' background. Compared with learners in HICs, learners in LMIC were less likely to have rural practice intentions at entry, but more likely at exit, suggesting that the power of these levers to strengthen the rural health workforce in LMICs might be even greater than in HICs (where the majority of studies to date have taken place). This is confirmed by other work coming from our partner schools in the Philippines (14) and Sudan (16). Further exploration of the cultural expectations of service and triangulation with placement experiences across settings may help to explore how these intentions change over the period of training and how strengthening the role of primary care within health systems could synergistically provide pathways and support for emerging health professionals.

Learners at exit were considerably more likely to intend a career in generalist disciplines, such as family medicine/general practice, than those at entry to their medical programs. Importantly, being female was associated with intention to practice in generalist disciplines and also with intention to practice in rural areas at exit, after adjusting for other learner characteristics and country income setting. These findings emphasize why community-based primary care focused training provided by these schools is so important to provide appropriate role models and inculcate the idea of service amongst their students (26). In the absence of these programs, it is difficult to have intentions to work in a discipline and geographic area to which there is no (positive) exposure, and with no clear profile or training pathway in the health system.

Many LMICs are experiencing challenges in implementing a primary health care approach as the role of family medicine, and training strategies and pathways are developed. Challenges include a low profile of family medicine, strengthening governance of primary care, unclear roles and responsibility for family medicine physicians, public mistrust in rural and primary health care providers, provision of appropriate training for the local context, limited infrastructure and supplies, and lack of evidence to inform policy makers (27, 28). While learners from LMICs in our study showed a high intent to practice in rural areas, a lack of policy support may pose significant challenges in realizing a rural workforce. Training in competencies and skills specifically for rural general practice has been incorporated into postgraduate training pathways for health professionals in HIC settings, and established to varying extents in LMIC settings. In Nepal and the Philippines for instance, efforts to support a PHC approach through postgraduate training programs in general practice/family medicine (21) have been challenging due to a lack of recognition of the value of primary health care in health policy, resulting in unclear career pathways and roles for general practitioners, and an overall effect of discouraging graduates from pursuing a generalist discipline. The Faculty of Medicine University of Gezira pioneered postgraduate training in family medicine in Sudan and is producing positive outcomes in rural practice. The program's first cohort of 207 candidates provided health services for 158 primary health care centers, most of them located in rural areas in Gezira State, of which 84 centers had never been served by a doctor (17). Recruitment of candidates to this program has been high reflecting the role of local universities in developing their communities however retention of the program's graduates in rural areas will become clearer in the future.

Interestingly, intention to work abroad was greater for learners at entry to the course than at exit (and although numbers are small at this stage of the prospective study, there was a significant shift to an intention to work in-country for learners with entry and exit data). This indicates a strong influence of educational experiences, both explicit and “hidden,” in strengthening a desire to serve the local region. High levels of emigration of doctors is an issue for LMICs however this downward trend in intention to work abroad was apparent for schools in both LMIC and HIC settings. A desire to gain experience motivated the majority of learners in LMICs who intended to work abroad at exit. Adding to this, a smaller proportion of learners in schools located in LMICs intended to work abroad for more than 10 years at exit, than at entry, suggesting that learners plan to return to the country of their medical school. These findings are particularly significant for LMIC countries, many of which have high rates of health worker emigration, few rural practitioners, and a privatized system that favors the wealthy. In addition, HICs have a responsibility to train an adequate workforce for their own rural areas, reducing dependence on international graduates (13).

This manuscript extends previous findings with a much larger cohort and matched data, allowing comparison between LMICs and HICs schools to assess the influence of the sociopolitical context of each school and the HRH planning context in the regions where the training takes place. There is increasing recognition that strengthening primary health care across LMICs is a critical component of strengthening health systems, particularly in rural areas where the population is dispersed, usually socio-economically disadvantaged and often more vulnerable to both communicable and non-communicable disease (2).

The strengths of this study are the inclusion of a large number of learners from a wide range of countries and contexts across five continents, collection of intention information at several time points, and the triangulation of contextual information about health systems and curricula with the survey data. Limitations however, include the fact that the majority of this data is still essentially two sets of cross-sectional data, with only 149 learners for whom matched entry and exit data is available to date and for whom true longitudinal information is available. In addition, given the many factors associated with choice of practice location, strong rural intent may not always result in later rural practice. Although early studies from some schools are encouraging (16), longitudinal studies with longer follow-up periods are important, especially given implications for future funding for rural initiatives (29). Furthermore, this study focused on learners' background characteristics and rural practice intent. It is the goal of socially accountable education to produce a workforce with a desire to serve the health needs of all underserved populations, including the urban underserved and those subsets of populations of lower socioeconomic status. Puddey et al. (30) found that graduates with a lower socioeconomic background were 1.63 times more likely to be practicing in areas of lower socioeconomic disadvantage, adding to the evidence for the need to select a diverse student body to meet the needs of underserved populations. Data from Ghent University were available but excluded for this analysis as rural practice was not considered to be sufficiently distinct from urban practice in Belgium.

## Conclusion

Understanding the drivers to serve rural and underserved regions amongst medical students and junior doctors is critical in producing a fit-for-purpose health workforce to provide universal coverage to primary health care and strengthened health equity in low and middle income countries. Understanding impacts across all levels in the rural training pathway is vital to optimize the chances of achieving this goal.

## Data Availability Statement

The datasets generated for this article are not readily available because access to the de-identified data is considered on request by all participating schools. Requests to access the datasets should be directed to Sarah Larkins, sarah.larkins@jcu.edu.au.

## Ethics Statement

This project has received ethical approval from the following bodies: James Cook University Human Research Ethics Committee, 4th November 2015, H6398 and then H7600. Ateneo De Zamboanga University Ethics Board Review, 2015. Flinders University, Social and Behavioral Research Ethics Committee (SBREC), 12th June 2014, Project Number 6387. Faculty of Medicine University of Gezira Ethical Committee. Lakehead University Research Ethics Board number 1465183, 20 May 2016, and Laurentian University Research Ethics Board number 6009708, 11 July 2016. Walter Sisulu University Faculty of Health Sciences Postgraduate, Training, Research and Ethics Unit, Human Research Committee, 3rd June 2014, Clearance 006/2014. Ethics Review Board, University of the Philippines, Manila. IRC, Patan Academy of Health Sciences. The patients/participants provided their written informed consent to participate in this study.

## Author Contributions

SL, A-JN, and FC conceived of the study with later input from SE, TW, JCH, and CL. KJ has been responsible for data storage and management and much of the analysis reported here. SL, KJ, MG, SE, CL, FC, SU, A-BO, TW, JCH, SK, and SL played major roles in driving local data collection in their settings and contributing to local data analysis. KJ and SL drafted the initial manuscript with input from all authors. All authors contributed to the article and approved the submitted version.

## Conflict of Interest

The authors declare that the research was conducted in the absence of any commercial or financial relationships that could be construed as a potential conflict of interest.
