# Supplementary material for: Training a Fit-For-Purpose Rural Health Workforce for Low- and Middle-Income Countries (LMICs): How Do Drivers and Enablers of Rural Practice Intention Differ Between Learners From LMICs and High Income Countries?
Source: Front Public Health. 2020 Oct 19;8:582464. doi: 10.3389/fpubh.2020.582464 (PMC7604342; doi:10.3389/fpubh.2020.582464)
Supplement: Supplementary file 1 [file Table_1.docx]

Supplementary Material 1

Predictors of intention to work in a rural location where binary variable is rural versus urban location at entry.^a^

|  | Number in unadjusted analysis | Unadjusted odds ratios  (95% CI; p-value) | Adjusted odds ratios  (95% CI; p-value) N=1574 |
| --- | --- | --- | --- |
| Increasing age | 3573 | 1.03 (1.02-1.05; 0.001) | 1.02 (0.99-1.05; 0.196) |
| LMIC school | 3598 | 0.87 (0.76-0.99; 0.033) | 0.68 (0.53-0.86; 0.002) |
| Female | 3592 | 1.26 (1.11-1.44; 0.001) | 1.17 (0.94-1.46; 0.154) |
| Income bottom two deciles | 2169 | 1.86 (1.54-2.26; <0.001) | 1.66 (1.29-2.14; <0.001) |
| Identify as underserved group | 3063 | 1.90 (1.61-2.25; <0.001) | 1.48 (1.13-1.93; 0.005) |
| Background - remote community | 361 | 5.83 (4.38-7.76; <0.001) | 6.09 (4.10-9.06; <0.001) |
| Background - small rural community | 326 | 5.91 (4.39-7.96; <0.001) | 6.32 (4.18-9.54; <0.001) |
| Background - regional centre or larger town | 505 | 3.48 (2.74-4.41; <0.001) | 4.05 (2.87-5.73; <0.001) |
| Background - major urban centre | 962 | 1.67 (1.38-2.04; <0.001) | 2.19 (1.64-2.94; <0.001) |
| Background - (major city/capital city; reference group) | 741 | N/A | N/A |

^a^ Rural quintiles (1=remote village, 2=small rural town, 3=large rural town) versus Urban quintiles (4=major regional centre and 5=major city or capital city). Excludes learners with an international background. CI=confidence interval.
