# Supplementary material for: Training a Fit-For-Purpose Rural Health Workforce for Low- and Middle-Income Countries (LMICs): How Do Drivers and Enablers of Rural Practice Intention Differ Between Learners From LMICs and High Income Countries?
Source: Front Public Health. 2020 Oct 19;8:582464. doi: 10.3389/fpubh.2020.582464 (PMC7604342; doi:10.3389/fpubh.2020.582464)
Supplement: Supplementary file 2 [file Table_2.docx]

Supplementary Material 2

Predictors of intention to work in a rural location where binary variable is rural versus urban location at exit.^a^

|  | Number in unadjusted analysis | Unadjusted odds ratios  (95% CI; p-value) | Adjusted odds ratios  (95% CI; p-value) N=597 |
| --- | --- | --- | --- |
| Increasing age | 1102 | 1.07 (1.04-1.10; <0.001) | 1.10 (1.04-1.15; <0.001) |
| LMIC school | 1135 | 1.50 (1.16-1.93; 0.002) | 1.71 (1.14-2.55; 0.009) |
| Female | 1132 | 1.51 (1.18-1.93; 0.001) | 1.73 (1.21-2.48; 0.003) |
| Income bottom two deciles | 790 | 1.09 (0.80-1.50; 0.576) | 0.87 (0.59-1.30; 0.507) |
| Identify as underserved group | 974 | 1.15 (0.83-1.58; 0.407) | 0.89 (0.57-1.40; 0.621) |
| Background - remote community | 115 | 3.59 (2.25-5.72); <0.001) | 3.23 (1.70-6.14; 0.001) |
| Background - small rural community | 129 | 3.23 (2.06-5.06); <0.001) | 3.82 (2.08-7.04; <0.001) |
| Background - regional centre or larger town | 133 | 2.37 (1.52-3.69); <0.001) | 2.48 (1.35-4.57; 0.004) |
| Background - major urban centre | 348 | 2.34 (1.64-3.33); <0.001) | 2.20 (1.28-3.77; 0.004) |
| Background - (major city/capital city; reference group) | 233 | N/A | N/A |

^a^ Rural quintiles (1=remote village, 2=small rural town, 3=large rural town) versus Urban quintiles (4=major regional centre and 5=major city or capital city). Excludes learners with an international background. CI=confidence interval.
