# Supplementary material for: Training a Fit-For-Purpose Rural Health Workforce for Low- and Middle-Income Countries (LMICs): How Do Drivers and Enablers of Rural Practice Intention Differ Between Learners From LMICs and High Income Countries?
Source: Front Public Health. 2020 Oct 19;8:582464. doi: 10.3389/fpubh.2020.582464 (PMC7604342; doi:10.3389/fpubh.2020.582464)
Supplement: Supplementary file 3 [file Table_3.docx]

Supplementary Material 3

Predictors of intention to work in a generalist discipline where binary variable is “family medicine/general practice” and “other discipline” at entry and exit. (Unsure option removed from analysis)

|  | Entry | Exit |
| --- | --- | --- |
|  | Adjusted odds ratios  (95% CI; p-value) (n=1274) | Adjusted odds ratios  (95% CI; p-value) (n=549) |
| Increasing age | 1.10 (1.07-1.14; <0.001) | 1.05 (0.99-1.11; 0.097) |
| Female | 0.93 (0.67-1.29; 0.667) | 2.10 (1.30-3.39; 0.002) |
| LMIC school | 0.19 (0.12-0.28; <0.001) | 0.15 (0.08-0.28; <0.001) |
| Bottom two deciles | 1.16 (0.80-1.70; 0.437) | 0.75 (0.45-1.24; 0.255) |
| Identify as underserved group | 1.38 (0.92-1.05; 0.116) | 1.36 (0.77-2.41; 0.292) |
| Rural background (Quintiles 1, 2 and 3) | 2.05 (1.47-2.86; <0.001) | 1.51 (0.98-2.33; 0.063) |

Excludes learners with an international background. CI=confidence interval.
