# Supplementary material for: Training a Fit-For-Purpose Rural Health Workforce for Low- and Middle-Income Countries (LMICs): How Do Drivers and Enablers of Rural Practice Intention Differ Between Learners From LMICs and High Income Countries?
Source: Front Public Health. 2020 Oct 19;8:582464. doi: 10.3389/fpubh.2020.582464 (PMC7604342; doi:10.3389/fpubh.2020.582464)
Supplement: Supplementary file 4 [file Table_4.docx]

Supplementary Material 4

Predictors of intention to work abroad where binary variable is “yes – intend to work abroad” and “No – don’t intend to work abroad” at entry. (Unsure option removed from analysis)

|  | Number in unadjusted analysis | Unadjusted odds ratios  (95% CI; p-value) | Adjusted odds ratios  (95% CI; p-value) (n=1084) |
| --- | --- | --- | --- |
| Increasing age | 2443 | 0.89 (0.87-0.90; <0.001) | 0.90 (0.87-0.92; <0.001) |
| LMIC school | 2485 | 0.76 (0.64-0.89; 0.001) | 0.82 (0.60-1.11; 0.192) |
| Female | 2455 | 1.04 (0.88-1.23; 0.685) | 0.90 (0.69-1.17; 0.433) |
| Income top two deciles | 1504 | 1.59 (1.28-1.97; <0.001) | 1.07 (0.79-1.44; 0.680) |
| Does not identify as underserved group | 2070 | 3.27 (2.66-4.02; <0.001) | 2.31 (1.71-3.13; <0.001) |
| Urban background (Quintiles 4 and 5) | 1971 | 1.29 (1.08-1.55; 0.006) | 1.44 (1.11-1.87; 0.007) |

Excludes learners with an international background. CI=confidence interval.
