# Supplementary material for: Training a Fit-For-Purpose Rural Health Workforce for Low- and Middle-Income Countries (LMICs): How Do Drivers and Enablers of Rural Practice Intention Differ Between Learners From LMICs and High Income Countries?
Source: Front Public Health. 2020 Oct 19;8:582464. doi: 10.3389/fpubh.2020.582464 (PMC7604342; doi:10.3389/fpubh.2020.582464)
Supplement: Supplementary file 5 [file Table_5.docx]

Supplementary Material 5

Predictors of intention to work abroad where binary variable is “yes – intend to work abroad” and “No – don’t intend to work abroad” at exit. (Unsure option removed from analysis)

|  | Number in unadjusted analysis | Unadjusted odds ratios  (95% CI; p-value) | Adjusted odds ratios  (95% CI; p-value) (n=411) |
| --- | --- | --- | --- |
| Increasing age | 759 | 0.83 (0.79-0.87; <0.001) | 0.85 (0.80-0.91; <0.001) |
| LMIC school | 778 | 0.30 (0.22-0.42; <0.001) | 0.22 (0.13-0.36; <0.001) |
| Female | 776 | 1.10 (0.83-1.47; 0.515) | 1.35 (0.85-2.14; 0.198) |
| Income top two deciles | 538 | 2.50 (1.75-3.56; <0.001) | 1.28 (0.79-2.09; 0.318) |
| Does not identify as underserved group | 647 | 2.77 (1.84-4.15; <0.001) | 1.54 (0.86-2.76; 0.144) |
| Urban background (Quintiles 4 and 5) | 649 | 0.90 (0.66-1.23; 0.501) | 1.11 (0.71-1.75; 0.640) |

Excludes learners with an international background. CI=confidence interval.
